# Honokiol Ameliorates LPS/D-GalN-Induced Acute Liver Failure via Activation of SIRT3/AMPK and Keap1/Nrf2/HO-1 Signaling and Inhibition of the NF-κB/NLRP3 Inflammasome Axis

**DOI:** 10.3390/ph19060909

**Published:** 2026-06-08

**Authors:** Abdulaziz F. Alhussaini, Mahmoud Elshal, Sara H. Hazem

**Affiliations:** 1 Department of Pharmacology and Toxicology, Faculty of Pharmacy, Mansoura University, ElGomhoria Street, Mansoura 35516, Egypt; 2 AlGhad College for Applied Medical Sciences, Najran 66243, Saudi Arabia

**Keywords:** honokiol, hepatoprotection, acute liver failure, LPS/D-galactosamine, oxidative stress, inflammation

## Abstract

**Background/Objectives:** Abrupt impairment of liver functions, extensive hepatocellular necrosis, and high short-term mortality in patients without pre-existing liver disease are characteristic hallmarks of acute liver failure (ALF). Therapeutic options for ALF remain extremely limited, with liver transplantation representing the only definitive intervention in advanced cases, highlighting the urgent need for effective pharmacological strategies to limit early hepatic injury and disease progression. Honokiol (HON), a biphenolic lignan derived from Magnolia species, possesses documented antioxidant and anti-inflammatory properties; however, its protective potential and mechanisms in ALF remain incompletely defined. **Methods:** A lipopolysaccharide (LPS)/D-galactosamine (D-GalN)-induced ALF murine model was employed to explore the hepatoprotective potential of HON (5 and 10 mg/kg) and its possible underlying mechanisms. **Results:** HON pretreatment significantly (i) attenuated hepatocellular injury, as evidenced by marked reductions in serum liver enzyme levels and suppression of necroinflammation and neutrophil infiltration. HON also (ii) restored hepatic redox homeostasis by enhancing antioxidant defenses and reducing lipid peroxidation and nitrosative stress. These effects were accompanied by (iii) activation of the Keap1/Nrf2/HO-1 signaling axis; (iv) suppression of NF-κB activation with subsequent inhibition of NLRP3 inflammasome and caspase-1 activation; and (v) reduction in hepatic TNF-α, IL-1β, and IL-18 levels. In parallel, HON (vi) restored mitochondrial SIRT3 and AMPK signaling. **Conclusions:** Our findings demonstrate that HON effectively protects against LPS/D-GalN-induced ALF via coordinated enhancement of antioxidant defense and suppression of inflammatory signaling pathways, supporting its potential as a promising pleiotropic therapeutic candidate for ALF.

## 1. Introduction

Acute liver failure (ALF) is characterized by rapid deterioration in hepatic function and extensive hepatocellular injury, which is associated with acute fulminant hepatitis [1]. ALF arises from autoimmune, viral, ischemic, and iatrogenic etiologies, besides malignancy and a range of less frequent causes [2]. Despite advances in intensive care, survival without liver transplantation remains poor, highlighting the need for mechanistically targeted pharmacological approaches that can prevent or attenuate fulminant hepatic injury [3].

Among available experimental systems, the lipopolysaccharide/D-galactosamine (LPS/D-GalN) model in rodents is widely used because it reproduces key histological and biochemical features of human ALF [4]. D-GalN depletes uridine nucleotides in hepatocytes and sensitizes the liver to the pro-inflammatory and pro-apoptotic actions of the endotoxin LPS [5]. This model engages Toll-like receptor (TLR)-mediated nuclear factor (NF)-κB activation, with subsequent reactive oxygen species (ROS) overproduction, pro-inflammatory cytokine release, and NOD-like receptor family pyrin domain-containing 3 (NLRP3) inflammasome activation [6,7].

In view of this injurious LPS/TLR, NF-κB, and NLRP3 axis, attention has turned to endogenous cytoprotective pathways that counter oxidative stress and restrict inflammatory signaling. AMP-activated protein kinase (AMPK), a critical sensor of intracellular energy levels, coordinates responses to metabolic stress and can limit oxidative injury through effects on mitochondrial function and downstream stress pathways [8]. Sirtuins, particularly the mitochondrial deacetylase sirtuin-3 (SIRT3), further modulate mitochondrial bioenergetics, ROS production, and cell survival [9].

The nuclear factor erythroid 2-related factor 2 (Nrf2) transcription factor also has a key regulatory role in oxidative stress and inflammatory responses to various insults, including LPS/D-GalN. Inactive Nrf2 is kept in the cytoplasm by Kelch-like ECH-associated protein 1 (Keap1). Upon oxidative stress or inflammation, dissociation of the Nrf2/Keap1 complex occurs, and subsequent nuclear translocation of Nrf2 takes place to activate the transcription of different enzymes, including heme oxygenase (HO)-1 [10].

Honokiol (HON, 5,3′-diallyl-2,4′-dihydroxybiphenyl) is a small biphenolic lignan mainly isolated from the bark, seed cones, and leaves of Magnolia species, particularly *Magnolia officinalis*. It exhibits antioxidant, anti-inflammatory, and hepatoprotective activities, with a favorable toxicological profile in preclinical systems. From a technical perspective, HON is considered a promising multitarget phytochemical due to its capacity to simultaneously modulate interconnected oxidative stress and inflammatory signaling cascades [11,12]. Previous studies reported that HON modulates NF-κB signaling [13] and acts as a potent ROS scavenger and a regulator of redox-sensitive signaling [14]. In hepatic models of oxidative injury, HON ameliorated oxidative damage in hepatocytes by inducing SIRT3 signaling [15]. HON was also reported to mitigate steatotic injury induced by a methionine-choline-deficient diet [16] and ameliorate lipotoxicity in non-alcoholic fatty liver disease by inducing SIRT3/AMPK signaling [17]. Moreover, in LPS-induced acute lung injury, HON has been reported to repress NLRP3 inflammasome-mediated pyroptosis through Nrf2 activation [18].

Accordingly, HON has been highlighted as a candidate small molecule for targeted modulation of oxidative and inflammatory signaling. However, the ability of HON to influence the rapid, severe hepatocellular injury characteristic of LPS/D-GalN-induced ALF remains incompletely defined. We therefore investigated whether HON could mitigate LPS/D-GalN-induced ALF in mice and characterize the downstream effectors responsible for such an effect.

## 2. Results

### 2.1. Effect of HON on Serum Biochemical Indices of Hepatic Injury in LPS/D-GalN-Challenged Mice

**Figure 1:**
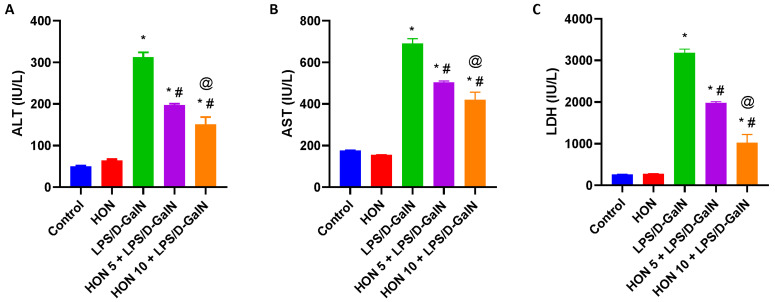
Effect of honokiol (HON) pretreatment on serum biochemical indices of hepatic injury in lipopolysaccharide (LPS)/D-galactosamine (D-GalN)-challenged mice. (**A**) Alanine aminotransferase (ALT), (**B**) Aspartate aminotransferase (AST), and (**C**) Lactate dehydrogenase (LDH) serum activities. Data are expressed as mean ± SEM (n = 6). *, #, @ significant difference compared to the control, LPS/D-GalN, HON 5 + LPS/D-GalN groups, respectively.

The LPS/D-GalN challenge produced marked hepatocellular injury, evidenced by highly significant increases in serum transaminases (ALT and AST) and LDH relative to the control group (Figure 1A–C, respectively). Relative to the control mice, ALT, AST, and LDH activities increased by about 6.4, 3.9, and 12.3-fold, respectively, in the LPS/D-GalN-challenged mice. Nevertheless, HON pretreatment attenuated the LPS/D-GalN-induced rise in all serum injury indices. Compared with the LPS/D-GalN group, HON (5 mg/kg) pretreatment significantly reduced ALT, AST, and LDH activities by about 37.1%, 27.4%, and 37.4%, respectively. Otherwise, the higher dose produced a stronger effect, where HON (10 mg/kg) reduced ALT, AST, and LDH by about 54.9%, 42.5%, and 71.3%, respectively, relative to the LPS/D-GalN group, indicating a dose-related effect of HON on these liver injury biomarkers. Notably, HON alone did not produce any significant alterations in ALT, AST, and LDH activities relative to the control group.

### 2.2. Effect of HON on Hepatic Oxidative and Nitrosative Imbalance in LPS/D-GalN-Challenged Mice

**Figure 2:**
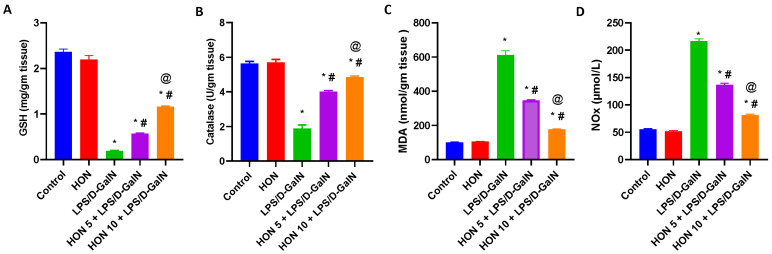
Effect of honokiol (HON) on hepatic oxidative and nitrosative stress markers in lipopolysaccharide (LPS)/D-galactosamine (D-GalN)-challenged mice. (**A**) Reduced glutathione (GSH), (**B**) Catalase activity, (**C**) Malondialdehyde (MDA), and (**D**) Total nitrite/nitrate (NOx) levels. Data are expressed as mean ± SEM (n = 6). *, #, @ significant difference compared to the control, LPS/D-GalN, HON 5 + LPS/D-GalN groups, respectively.

LPS/D-GalN significantly disrupted hepatic redox homeostasis, with a profound depletion of GSH content and catalase activity and marked increases in lipid peroxidation and nitric oxide (NO) production compared with the control group (Figure 2A–D, respectively). Relative to the control group, hepatic GSH fell to 0.09-fold and catalase fell to 0.33-fold, whereas MDA increased by about 6.2-fold and NO increased by 3.9-fold. Otherwise, HON alone did not yield significant alterations compared with the control group.

HON pretreatment significantly counteracted the oxidant and nitrosative changes induced by LPS/D-GalN. Relative to the LPS/D-GalN group, HON (5 mg/kg) significantly increased GSH by about 496.9% and catalase by 115.8%, while reducing MDA by 44.1% and NO by 37.4% (Figure 2A–D, respectively). Meanwhile, HON (10 mg/kg) produced a more pronounced improvement and increased GSH by about 184.1% and catalase by 161%, while decreasing MDA by 71.6% and NO by 62.8%, with high significance relative to the LPS/D-GalN group. Notably, the effect of HON on the above biomarkers was dose-related.

### 2.3. Effect of HON on Hepatic Histopathological Alterations in LPS/D-GalN-Challenged Mice

**Figure 3:**
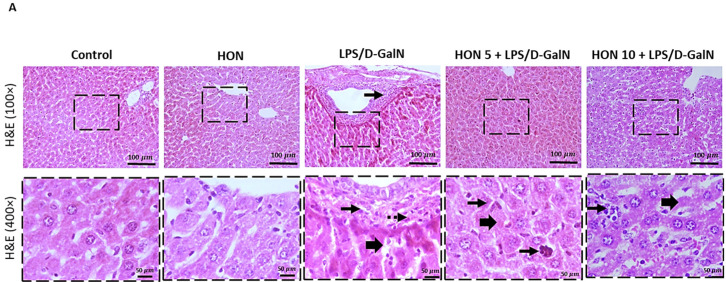

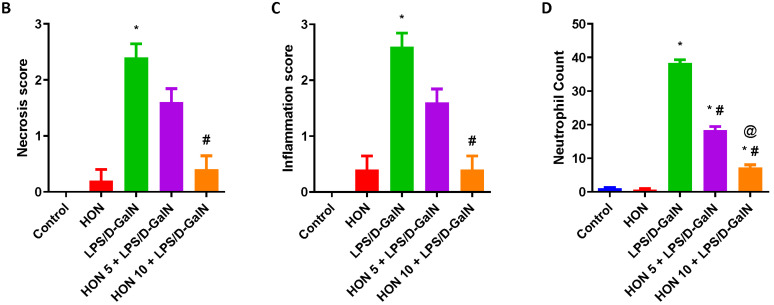
Effect of honokiol (HON) on hepatic histopathological alterations in lipopolysaccharide (LPS)/D-galactosamine (D-GalN)-challenged mice. (**A**) Representative hematoxylin and eosin (H&E)-stained liver sections showing preserved hepatic architecture in the control and HON-treated groups, whereas the LPS/D-GalN group demonstrates marked hepatocellular necrosis and inflammatory infiltration that were attenuated following HON pretreatment. Thin arrows, inflammation; thick arrows, hepatocellular necrosis; dashed thin arrow, fibrosis. Upper panel magnification ×100, scale bar = 100 µm; lower panel magnification ×400, scale bar = 20 µm, (**B**) Corresponding necrosis score, (**C**) Corresponding inflammatory score, and (**D**) Corresponding neutrophil count. Data are expressed as mean ± SEM (n = 4). *, #, @ significant difference compared to the control, LPS/D-GalN, HON 5 + LPS/D-GalN groups, respectively.

As shown in Figure 3A, H & E-stained liver tissue sections showed preserved hepatic tissue integrity in the control and HON alone groups. In contrast, the LPS/D-GalN induced histopathological abnormalities, including extensive hepatocellular injury with marked necroinflammation and inflammatory cell infiltration that were suppressed with HON pretreatment, especially with the higher dose (10 mg/kg).

These necrosis and inflammation scores, as well as the infiltrated neutrophil count, were addressed for further illustration (Figure 3B–D, respectively). Our findings demonstrated significantly higher necrosis and inflammation scores and neutrophil count in the LPS/D-GalN group relative to the control group. Upon HON pretreatment, the necrosis score was reduced by about 33.3% and 83.3% in the 5 mg/kg and 10 mg/kg HON-treated groups, respectively, showing a significant reduction in the 10 mg/kg-treated group relative to the LPS/D-GalN group. Similarly, HON pretreatment reduced necrosis severity by about 2.2 and 6-fold in the 5 mg/kg and 10 mg/kg-treated groups, when compared to the disease control group. Regarding inflammatory cell infiltration, HON pretreatment reduced neutrophil count by 18.4-fold with the 5 mg/kg dose and by 7.2-fold with the 10 mg/kg dose, relative to the LPS/D-GalN group, with a greater decrease at the higher dose. Notably, administration of HON alone did not show any significant effect on any of the aforementioned parameters compared to the control group. Therefore, the HON group was excluded from subsequent molecular mechanistic analyses.

### 2.4. Effect of HON on the Nrf2 Signaling in the Liver in LPS/D-GalN-Challenged Mice

**Figure 4:**
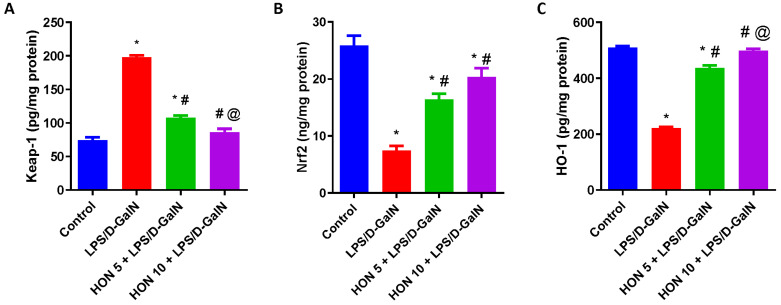
Effect of honokiol (HON) on hepatic Kelch-like ECH-associated protein 1 (Keap1)/nuclear factor erythroid 2-related factor 2 (Nrf2)/heme oxygenase-1 (HO-1) signaling pathway in lipopolysaccharide (LPS)/D-galactosamine (D-GalN)-challenged mice. (**A**) Keap1, (**B**) Nrf2, and (**C**) HO-1 protein levels. Data are expressed as mean ± SEM (n = 6). *, #, @ significant difference compared to the control, LPS/D-GalN, HON 5 + LPS/D-GalN groups, respectively.

LPS/D-GalN markedly altered the Keap1/Nrf2/HO-1 regulatory axis, consistent with suppression of the antioxidant power. Compared with the control group, Nrf2 protein levels decreased to about 0.29-fold, and HO-1 decreased to 0.43-fold, while Keap1 increased by about 2.7-fold (Figure 4A–C, respectively). Alternatively, HON pretreatment restored this axis. Compared with the disease control group, HON (5 mg/kg) significantly increased Nrf2 levels by about 119.9% and HO-1 by 97.7%, while reducing Keap1 by 45.5%. Meanwhile, HON (10 mg/kg) produced a greater restoration, where Nrf2 increased by about 172.1%, and HO-1 increased by 125.5%, with Keap1 reduced by about 56.5% compared with the disease control group. Notably, the 10 mg/kg dose of HON showed additional enhancement of Nrf2 signaling that was represented by significantly higher HO-1 levels and lower Keap1 levels compared with the lower dose.

### 2.5. Effect of HON on Pro-Inflammatory Cytokine Expression in the Liver in LPS/D-GalN-Challenged Mice

**Figure 5:**
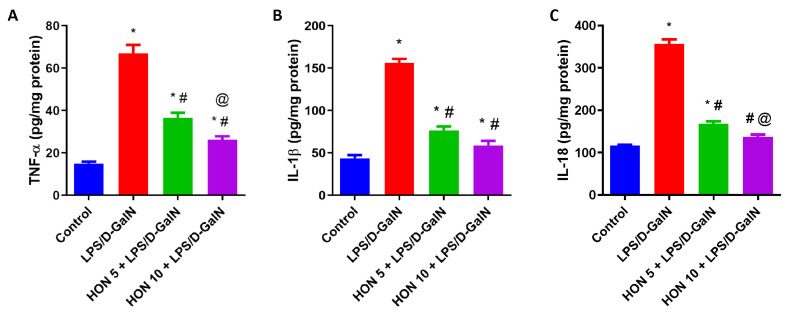
Effect of honokiol (HON) on hepatic pro-inflammatory cytokine levels in lipopolysaccharide (LPS)/D-galactosamine (D-GalN)-challenged mice. (**A**) Tumor necrosis factor alpha (TNF-α), (**B**) Interleukin (IL)-1β, (**C**) IL-18. Data are expressed as mean ± SEM (n = 6). *, #, @ significant difference compared to the control, LPS/D-GalN, HON 5 + LPS/D-GalN groups, respectively.

The LPS/D-GalN group showed significant elevation in hepatic TNF-α, IL-1β, and IL-18 compared with the control group (Figure 5A–C, respectively). TNF-α increased by about 4.5-fold, IL-1β increased by about 3.6-fold, and IL-18 increased by about 3.1-fold relative to the control group. Meanwhile, HON pretreatment significantly reduced these cytokines compared with the LPS/D-GalN group. HON (5 mg/kg) significantly reduced TNF-α by about 45.5%, IL-1β by about 51.1%, and IL-18 by about 52.8%, relative to the disease control group. Alternatively, HON (10 mg/kg) produced greater suppression, TNF-α decreased by about 60.9%, IL-1β decreased by about 62.3%, and IL-18 decreased by about 61.5% versus the disease control group. Dose-related effect of HON on TNF-α and IL-18 levels was noticed.

### 2.6. Effect of HON on Hepatic NF-κB Immunoexpression in LPS/D-GalN-Challenged Mice

**Figure 6:**
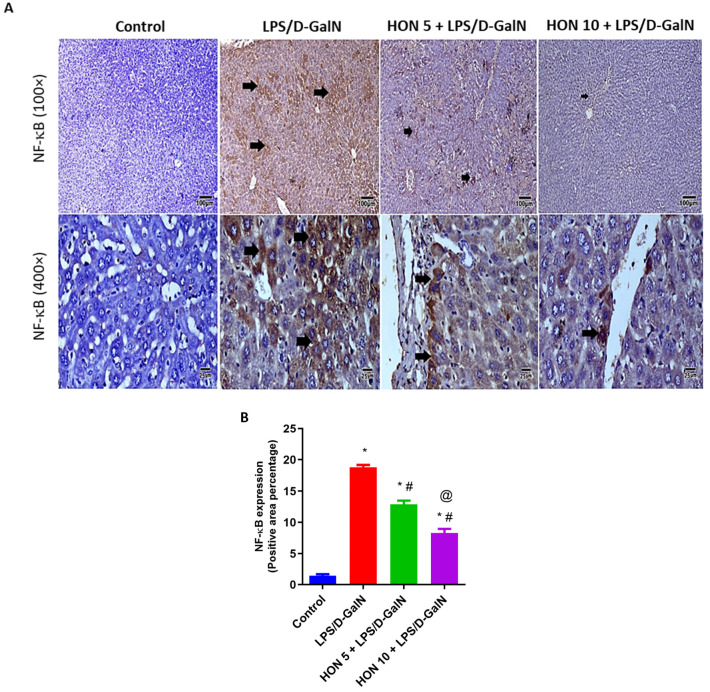
Effect of honokiol (HON) on hepatic nuclear factor (NF)-κB immunoexpression in lipopolysaccharide (LPS)/D-galactosamine (D-GalN)-challenged mice. (**A**) Representative immunohistochemical images showing NF-κB expression. LPS/D-GalN remarkably increased NF-κB staining compared to the control group. HON pretreatment markedly reduced NF-κB expression compared to the LPS/D-GalN group, and (**B**) Corresponding percentage of positive NF-κB expression area. Data are expressed as mean ± SEM (n = 4). *, #, @ significant difference compared to the control, LPS/D-GalN, HON 5 + LPS/D-GalN groups, respectively.

Photomicrographs of mice liver tissue sections from the control group show negative NF-κB p65 immunostaining (Figure 6A). In contrast, sections from the LPS/D-GalN group showed marked NF-κB p65 immunostaining (thick arrows). Alternatively, the 5 mg/kg-treated group revealed moderate NF-κB p65 immunostaining, while the high dose-treated group showed minimal NF-κB p65 immunostaining. As illustrated in Figure 6B, LPS/D-GalN challenge induced a significant elevation in NF-κB p65 expression in the liver by nearly 12.8-fold relative to the control group. HON alone did not induce a significant activation in NF-κB p65 expression relative to the control group. Meanwhile, HON pretreatment significantly attenuated the LPS/D-GalN-induced hepatic NF-κB p65 activation in a dose-related manner. Low-dose HON group (5 mg/kg) reduced NF-κB p65 expression by about 31.3% relative to the LPS/D-GalN group. On the other hand, the high-dose group (10 mg/kg) produced a greater suppression, reducing NF κB p65 by about 55.9% relative to the LPS/D-GalN group.

### 2.7. Effect of HON on SIRT3/NLRP3 Signaling in the Liver in LPS/D-GalN-Challenged Mice

**Figure 7:**
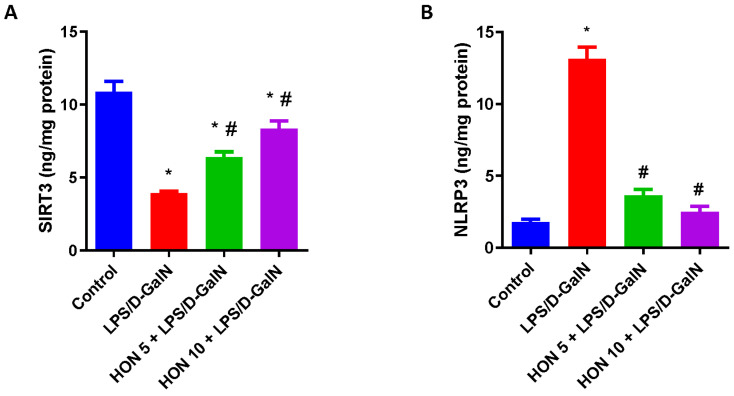
Effect of honokiol (HON) on hepatic sirtuin-3 (SIRT3) and NOD-like receptor family pyrin domain-containing 3 (NLRP3) signaling in lipopolysaccharide (LPS)/D-galactosamine (D-GalN)-challenged mice. (**A**) SIRT3 and (**B**) NLRP3 protein expression levels. Data are expressed as mean ± SEM (n = 6). *, # significant difference compared to the control and LPS/D-GalN groups, respectively.

LPS/D-GalN significantly reduced hepatic SIRT3 and increased NLRP3 relative to the control group (Figure 7A and Figure 7B, respectively). SIRT3 declined to about 0.36-fold, whereas NLRP3 increased by about 7.4-fold versus the control group. In contrast, HON pretreatment significantly counteracted both alterations. Compared with the untreated LPS/D-GalN group, the lower dose of HON (5 mg/kg) markedly increased SIRT3 by about 62.6% and reduced NLRP3 by about 72.3%. Otherwise, HON (10 mg/kg) produced a stronger response, increasing SIRT3 by about 112.7% and reducing NLRP3 by about 81.1%.

### 2.8. Effect of HON on Hepatic Caspase-1 Activation in LPS/D-GalN-Challenged Mice

**Figure 8:**
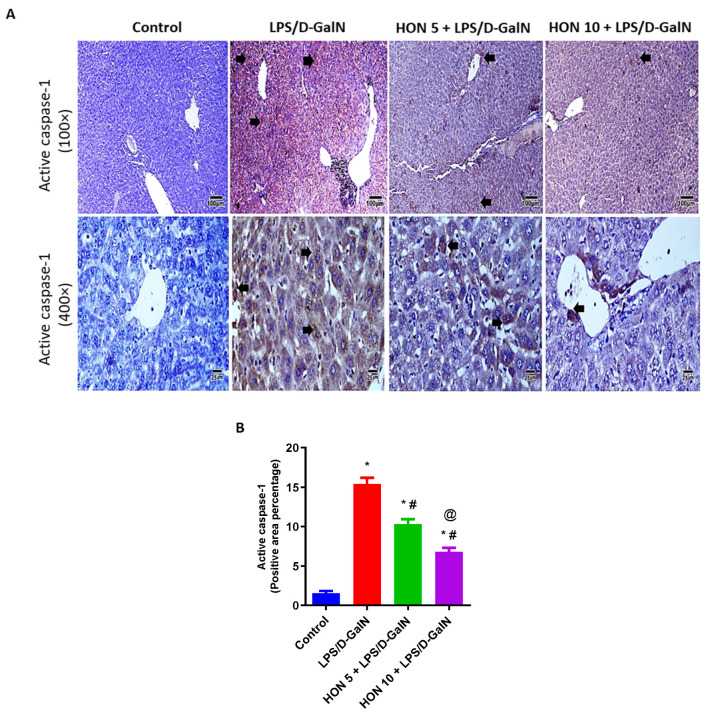
Effect of honokiol (HON) on hepatic active caspase-1 immunoexpression in lipopolysaccharide (LPS)/D-galactosamine (D-GalN)-challenged mice. (**A**) Representative immunohistochemical images showing active caspase-1 expression. LPS/D-GalN significantly increased caspase-1 staining compared to the control group. HON pretreatment markedly reduced active caspase-1 expression compared to the LPS/D-GalN group, and (**B**) Corresponding percentage of positive active caspase-1 expression area. Data are expressed as mean ± SEM (n = 4). *, #, @ significant difference compared to the control, LPS/D-GalN, HON 5 + LPS/D-GalN groups, respectively.

As presented in Figure 8A, illustrative micrographs of hepatic sections from the control group show negative active caspase-1 immunostaining, while marked active caspase-1 expression (thick arrows) is observed in the LPS/D-GalN group. In contrast, the HON 5 + LPS/D-GalN group showed moderate active caspase-1 immunostaining, and the HON 10 + LPS/D-GalN group demonstrated minimal active caspase-1 immunostaining. In addition, the LPS/D-GalN challenge induced a significant elevation in the hepatic abundance of active caspase-1 by about 9.9-fold relative to the control group (Figure 8B). HON alone did not induce a significant activation in active caspase-1 expression when compared to the control group. Meanwhile, HON pretreatment significantly attenuated the LPS/D-GalN hepatic caspase-1 activation. The low-dose HON group (5 mg/kg) reduced active caspase-1 expression by about 33.1% relative to the LPS/D-GalN group. In contrast, the high-dose group (10 mg/kg) produced a greater suppression, reducing active caspase-1 expression by about 55.88% relative to the LPS/D-GalN group. Notably, the effect of HON pretreatment on caspase-1 activation in the liver was dose-related.

### 2.9. Effect of HON on AMPKα Activation in the Liver in LPS/D-GalN-Challenged Mice

**Figure 9:**
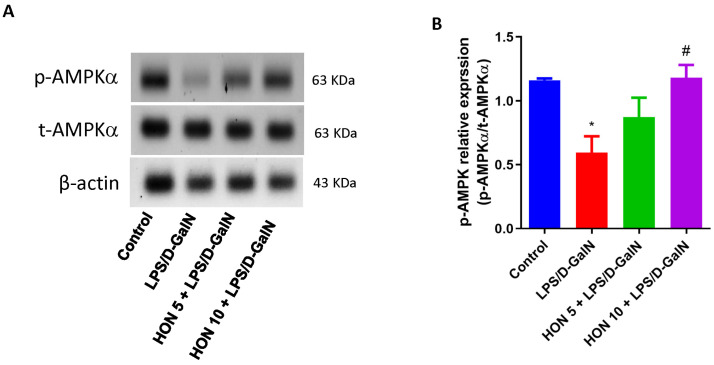
Effect of honokiol (HON) on hepatic AMP-activated protein kinase alpha (AMPKα) activation in lipopolysaccharide (LPS)/D-galactosamine (D-GalN)-challenged mice. (**A**) Representative Western blot images showing phosphorylated AMPKα (p-AMPKα), total AMPKα (t-AMPKα), and β-actin expression. (**B**) Relative expression ratio of p-AMPKα to t-AMPKα. Data are expressed as mean ± SEM (n = 3). *, # significant difference compared to the control and LPS/D-GalN groups, respectively.

As illustrated in Figure 9A, the protein abundance ratio of phosphorylated AMPKα to total AMPKα is reduced following the LPS/D-GalN challenge, whereas HON pretreatment nearly restored it. LPS/D-GalN significantly reduced the phosphorylated AMPKα to total AMPKα protein ratio compared with the control group by about 0.2-fold (Figure 9B). However, HON (5 mg/kg) pretreatment increased the p-AMPKα to total AMPKα ratio by about 271.8%; only the higher dose of HON (10 mg/kg) significantly increased AMPKα activation by about 320% relative to the LPS/D-GalN group.

## 3. Discussion

The current study provides the initial evidence that HON confers robust protection against LPS/D-GalN-induced ALF via coordinated antioxidant and anti-inflammatory actions. Importantly, these protective activities were dose-related and mechanistically associated with the regulation of the Keap1/Nrf2/HO-1 axis, suppression of NF-κB-driven inflammatory signaling, attenuation of NLRP3 inflammasome activation, and restoration of mitochondrial SIRT3/AMPK signaling.

Oxidative stress constitutes a central initiating event in LPS/D-GalN-induced liver damage, driven by excessive ROS generation from activated Kupffer cells and recruited neutrophils, coupled with depletion of endogenous antioxidant defenses [19,20]. In agreement with previous reports, LPS/D-GalN intoxication in the ongoing study produced profound disruption of hepatic redox homeostasis, evidenced by marked depletion of GSH and catalase activity alongside excessive lipid peroxidation products and NO production. These biochemical disturbances were accompanied by severe hepatocellular injury, reflected by marked elevations in serum biomarkers of liver injury. HON pretreatment markedly restored antioxidant capacity, suppressed lipid peroxidation and nitrosative stress, and significantly reduced serum liver enzyme release in a dose-related manner, indicating effective interruption of the initial oxidative injury phase. These findings are consistent with earlier observations that HON acts as a potent ROS scavenger and mitochondrial stabilizer in diverse models of hepatic and extrahepatic injury [20,21].

At the molecular level, restoration of redox balance by HON strongly coincided with reactivation of the Keap1/Nrf2/HO-1 antioxidant signaling. LPS/D-GalN challenge markedly downregulated hepatic Nrf2 and HO-1 expression while elevating Keap1, reflecting impaired antioxidant transcriptional responses. HON pretreatment reversed these changes in a dose-related manner. Activation of this pathway is widely recognized as a critical protective mechanism in experimental ALF, limiting oxidative stress and restraining inflammatory amplification [22,23]. These findings align with previous studies demonstrating that pharmacological Nrf2 activation mitigates the LPS/D-GalN-induced hepatic damage and improves survival outcomes [24,25,26]. Previously, it was reported that HON activates Nrf2, mitigating LPS-induced acute lung injury [18] and metabolic-associated fatty liver disease [20].

Beyond oxidative stress, uncontrolled inflammation represents a dominant driver of ALF progression. TNF-α, IL-1β, and IL-18 are pivotal cytokines that amplify hepatocellular injury through the recruitment of inflammatory cells and activation of inflammasome pathways [27,28]. In the ongoing study, LPS/D-GalN produced a pronounced elevation of these cytokines, accompanied by marked neutrophil infiltration and extensive histological necroinflammation. HON pretreatment significantly suppressed hepatic TNF-α, IL-1β, and IL-18 levels, decreased inflammatory cell infiltration, and attenuated necrosis severity, with superior efficacy observed at the higher dose. These findings support a key anti-inflammatory role for HON in limiting the second phase of inflammation-driven liver injury. Previously, HON was shown to suppress the release of pro-inflammatory cytokines, including TNF-α, thereby influencing the inflammatory cell infiltration and improving the inflammatory response [29].

NF-κB signaling orchestrates the inflammatory cascade triggered by LPS/D-GalN. Activation of NF-κB stimulates transcription of TNF-α, IL-1β, IL-18, and iNOS, thereby sustaining cytokine release and NO overproduction [30,31,32]. In the present study, LPS/D-GalN provoked marked activation of hepatic NF-κB p65, while HON pretreatment significantly suppressed NF-κB nuclear immunoexpression in a dose-related manner. This suppression paralleled reductions in pro-inflammatory cytokines and NO levels, indicating that inhibition of NF-κB-dependent inflammatory signaling constitutes a major mechanism underlying HON-mediated hepatoprotection. Similar NF-κB inhibitory effects have been reported for HON in different inflammatory models, reinforcing the translational relevance of this pathway [30,33,34].

Emerging evidence highlights the importance of mitochondrial regulators in determining hepatocyte survival during ALF. SIRT3 serves as a cornerstone in maintaining mitochondrial homeostasis, limiting ROS generation, and suppressing inflammasome activation [35,36]. In the current study, LPS/D-GalN markedly suppressed hepatic SIRT3 expression, while HON pretreatment restored SIRT3 levels and suppressed NLRP3 inflammasome and caspase-1 activation, along with the attenuation of downstream inflammatory damage. These findings align with contemporary reports characterizing SIRT3 as a key negative regulator of NLRP3-mediated inflammation in hepatic injury models [37,38]. In parallel, HON significantly restored AMPK activation, which was profoundly suppressed by LPS/D-GalN challenge. AMPK functions as a cellular energy sensor and exerts hepatoprotective effects through modulation of mitochondrial metabolism, oxidative stress, and inflammatory signaling [8]. The stimulatory effect of HON on SIRT3 and AMPK was previously reported [9,17,39].

The current study has some limitations, as it establishes the protective potential of HON in an acute mouse model of LPS/D-GalN. However, the therapeutic efficacy of HON in chronic inflammatory settings is yet to be investigated. In addition, further studies are warranted to explore HON’s translational potential, optimal dosing strategies, and therapeutic window in clinically relevant settings. The study would also benefit from an in-silico study investigating possible interactions between different targeted genes and HON.

## 4. Materials and Methods

### 4.1. Drugs and Chemicals

HON was purchased as a pure powder from DC Chemicals (Shanghai, China) and freshly prepared as a sterile suspension in 0.5% *w*/*v* carboxymethylcellulose (CMC). LPS from Escherichia coli serotype O111:B4 and D-GalN were obtained from Sigma-Aldrich Chemical Co. (Saint Louis, MO, USA). LPS was reconstituted in sterile saline to 1 mg/mL, aliquoted, stored at −20 °C, and diluted immediately before administration. D-GalN was dissolved in sterile saline (0.9%) to a concentration of 70 mg/mL with gentle warming not exceeding 37 °C. All additional chemicals and reagents used in this investigation were of the highest commercially available grade.

### 4.2. Kits and Antibodies

Comprehensive, detailed information about kits and antibodies utilized in this investigation is documented in Supplementary Tables S1 and S2, respectively.

### 4.3. Animals

Adult male BALB/c mice (approximately 25 g) were secured from VACSERA and kept in standard plastic cages under controlled environmental conditions at a constant temperature of 25 °C with a 12 h/12 h light–dark cycle. Animals freely accessed a standard pellet diet and water throughout the experimental period. All animal handling, maintenance, and disposal procedures complied with the National Institutes of Health Guide for the Care and Use of Laboratory Animals [40]. The experimental protocol was reviewed and approved by the Mansoura University Animal Care and Use Committee with code number: MU-ACUC (PHARM.MS.24.09.108).

### 4.4. Experimental Design

Thirty mice were randomly allocated into five experimental groups (n = 6 per group) as follows: (1) Control group: received no treatments; (2) HON group: received HON only at the higher dose level (10 mg/kg); (3) LPS/D-GalN group: served as diseased group and received intraperitoneal (i.p.) injection of LPS (10 µg/kg) and D-GalN (700 mg/kg); (4) HON 5 + LPS/D-GalN: served as low-dose treated group and received HON (5 mg/kg, i.p.) dissolved in 0.5% *w*/*v* CMC once daily for four consecutive days, followed on day four by LPS and D-GalN injection one hour after the final HON dose; (5) HON 10 + LPS/D-GalN: served as high-dose treated group and received HON (10 mg/kg, i.p.) for four successive days and was similarly intoxicated with LPS and D-GalN. Dose selection was based on established murine models of D-GalN/LPS-induced hepatic injury [41] and previously validated HON regimens [42,43].

### 4.5. Sample Collection and Preparation

Following LPS/D-GalN administration on day four, mice were monitored for eight hours prior to anesthesia induction using thiopental sodium (70 mg/kg, i.p.) [44]. The sacrifice time point was determined at 8 h post-LPS/D-GalN to avoid spontaneous mortality and to capture the peak of oxidative stress and inflammatory response. Blood was harvested by cardiac puncture and centrifuged at 1000× *g* for 10 min at 4 °C to collect serum samples, which were used for subsequent biochemical analyses. Animals were then sacrificed, and the liver was rapidly excised and washed with ice-cold saline. The right median lobe was isolated and fixed in 10% (*v*/*v*) neutral-buffered formalin for preparation of paraffin blocks used in histopathological and immunohistochemical examinations. The left median lobe was dissected and divided into two portions: one portion was stored at −80 °C for Western blotting, while the remaining portion was homogenized in phosphate-buffered saline (10% *w*/*v*) and centrifuged at 3000× *g* for 20 min at 4 °C to obtain the supernatant [45], preserved at −80 °C for further biochemical analyses and ELISA.

### 4.6. Assessment of Hepatic Function

Serum activities of alanine aminotransferase (ALT), aspartate aminotransferase (AST), and lactate dehydrogenase (LDH) were measured using specific colorimetric diagnostic kits (AGAPPE, Kochi, India), following the manufacturers’ instructions.

### 4.7. Assessment of Hepatic Redox Status

Oxidative stress and antioxidant defense parameters were assessed in hepatic tissue homogenates. Lipid peroxidation was evaluated by estimating the levels of malondialdehyde (MDA), while antioxidant status was assessed by determining reduced glutathione (GSH) content and catalase activity. Moreover, the total nitrite/nitrate (NOx) as a marker for nitric oxide production was quantified. All redox and antioxidant parameters were determined using colorimetric assay kits (Biodiagnostic, Giza Governorate, Egypt), following the manufacturers’ instructions.

### 4.8. Histopathological Assessment of the Liver

Liver tissue sections were prepared at a thickness of 4 μm from the paraffin blocks. A standard hematoxylin and eosin (H&E) staining protocol was performed on sections mounted on glass slides. Histological assessment focused on hepatocellular necroinflammation and inflammatory cell infiltration. Histopathological changes were evaluated in a blinded manner using a semi-quantitative component-based scoring system, adapted from the validated methodology described by Veteläinen et al. (2006) [46]. Briefly, necrosis and inflammatory changes were scored independently on a scale reflecting increasing severity (0 = absent; 1 = mild; 2 = moderate; 3 = marked), based on the extent and distribution of the lesions observed across multiple microscopic fields. A composite histopathology score was obtained by summation of the individual component scores for each specimen. For the neutrophil count, four fields with maximum aggregates of polymorphonuclear leukocytes with segmented nuclei were counted for each group. Images were taken using a ToupCam digital camera (XCAM1080PHA; 2.8 megapixels; Bury St. Edmunds, Suffolk, UK) and an Olympus^®^ microscope (CX23LEDRF; Hachioji, Tokyo, Japan) at magnifications of ×100 and ×400.

### 4.9. Immunohistochemistry

Immunohistochemical staining was performed to determine the hepatic NF-κB p65 and active caspase-1 expression levels. Following deparaffinization by xylene, liver sections were subjected to rehydration and processed according to standard immunohistochemical protocols before incubation with the corresponding primary antibodies. Stained sections were visualized and photographed using the ToupCam digital camera (XCAM1080PHA; Suffolk, UK) mounted on the Olympus^®^ CX23LEDRF microscope at ×100 and ×400 magnifications. Quantitative assessment was performed by determining the percentage of positively stained area in randomly selected microscopic fields for each section using ImageJ software (version 1.52a, NIH, Bethesda, MD, USA). All immunohistochemical analyses were conducted in a blinded manner.

### 4.10. ELISA Measurements

Tumor necrosis factor-alpha (TNF-α), interleukin (IL)-1β, IL-18, NLRP3, SIRT3, Nrf2, Keap1, and HO-1 levels were quantified in hepatic tissue supernatants using corresponding mouse sandwich ELISA kits, following the guidelines of the manufacturers.

### 4.11. Western Blot Analysis

Hepatic total proteins were isolated using RIPA lysis buffer containing protease and phosphatase inhibitor cocktails (Bio BASIC INC., Markham, ON, Canada). A commercially available Bradford protein assay kit was employed to quantify protein concentration (Bio BASIC INC., Markham, ON, Canada) in accordance with the manufacturer’s instructions. Equal protein amounts (20 µg per sample) were mixed with 2× Laemmli sample buffer and heated at 95 °C for 5 min to ensure complete protein denaturation prior to electrophoresis. Sodium dodecyl sulfate polyacrylamide gel electrophoresis (SDS-PAGE) was performed to resolve proteins using TGX Stain-Free™ FastCast™ acrylamide gels (Bio-Rad Laboratories, Hercules, CA, USA). Electrophoresis was carried out at 50 V for 20 min, followed by an increase to 100–150 V for approximately 1 h. Resolved proteins were electroplotted onto polyvinylidene difluoride (PVDF) membranes using a Trans-Blot Turbo transfer system (Bio-Rad Laboratories) at 25 V for 7 min. Membranes were then blocked in Tris-buffered saline containing Tween-20 (TBST) and 3% bovine serum albumin for 1 h at room temperature. The blots were incubated overnight at 4 °C with the appropriate primary antibodies, including antibodies against total AMPKα, phosphorylated AMPKα (Ser486), and β-actin as a loading control (Thermo Fisher Scientific, Waltham, MA, USA), diluted following the guidelines of the manufacturer’s protocol. Membranes were washed with TBST and subsequently incubated with horseradish peroxidase-linked secondary antibodies for 1 h at ambient temperature and subsequently rinsed with TBST. Protein bands were visualized using a chemiluminescent detection system (Clarity™ Western ECL substrate, Bio-Rad Laboratories, Hercules, CA, USA) and captured with a CCD camera-based imaging system. Densitometric analysis was performed using image analysis software, and target protein expression levels were normalized to β-actin.

### 4.12. Statistical Analysis

Data were statistically evaluated utilizing GraphPad Prism software version 8.0 (GraphPad Software Inc., Solana Beach, CA, USA). Data conforming to parametric assumptions were analyzed by one-way analysis of variance (ANOVA), followed by Tukey–Kramer post hoc multiple comparison testing, and are presented as mean ± standard error (SE). Nonparametric data, including scoring variables, were analyzed using the Kruskal–Wallis test with Dunn’s post hoc test for pairwise comparisons. A probability value of less than 0.05 was considered to indicate statistical significance.

## 5. Conclusions

In conclusion, the present study demonstrates that HON effectively protects against LPS/D-GalN-induced ALF through coordinated enhancement of antioxidant defense and suppression of inflammatory signaling pathways. These protective effects were associated with activation of the Keap1/Nrf2/HO-1 antioxidant pathway, suppression of NF-κB-driven inflammatory signaling, and restoration of the SIRT3-AMPK signaling axis. The simultaneous modulation of these pathways may explain the broad and effective hepatoprotection observed, particularly at the higher dose of HON. Collectively, these findings suggest that HON may serve as a promising pleiotropic therapeutic candidate for ALF.

## Data Availability

The original contributions presented in this study are included in the article/Supplementary Material. Further inquiries can be directed to the corresponding author.

## Supplementary Materials

The following supporting information can be downloaded at: https://www.mdpi.com/article/10.3390/ph19060909/s1; Table S1: Assay kits; Table S2: Antibodies; Figure S1: Raw Western blot images.
