# Supplementary material for: Honokiol Ameliorates LPS/D-GalN-Induced Acute Liver Failure via Activation of SIRT3/AMPK and Keap1/Nrf2/HO-1 Signaling and Inhibition of the NF-κB/NLRP3 Inflammasome Axis
Source: Pharmaceuticals (Basel). 2026 Jun 8;19(6):909. doi: 10.3390/ph19060909 (PMC13305958; doi:10.3390/ph19060909)
Supplement: Supplementary file 1 [file pharmaceuticals-19-00909-s001.zip › pharmaceuticals-4292060-supplementary.pdf]

Table S 1: Assay kits

Kit	Supplier	Catalog. No.
Mouse TNF-α (tumor necrosis factor alpha) ELISA Kit	MyBioSource, USA	MBS825075
Mouse Nrf2 (nuclear factor erythroid 2-related factor 2) ELISA Kit	CUSABIO, USA	CSB-E16188m
Mouse IL-18 (Interleukin 18) ELISA Kit	Elabscience, USA	E-EL-M0730
Mouse IL-1 Beta ELISA Kit	MyBioSource, USA	MBS175967
Mouse HO-1 (Heme Oxygenase 1) ELISA Kit	MyBioSource, USA	MBS267777
Mouse KEAP1 (Kelch Like ECH Associated Protein 1) ELISA Kit	ELK Biotechnology, USA	ELK8105
Mouse NLRP3 (Nod Like Receptor Pyrins-3) ELISA Kit	MyBioSource, USA	MBS7606270
Mouse SIRT3 (Sirtuin 3) ELISA Kit	ELK Biotechnology, USA	ELK7151
Glutathione Reduced (GSH) Kit	Bio-diagnostic, Egypt	GR 2511
Catalase Assay Kit	Bio-diagnostic, Egypt	CA 2517
Malondialdehyde (MDA) Assay Kit	Bio-diagnostic, Egypt	MD 2529
Superoxide Dismutase (SOD) Assay Kit	Bio-diagnostic, Egypt	SD 2521
Nitric Oxide (NO) Assay Kit	Bio-diagnostic, Egypt	NO 2533
Lactate dehydrogenase (LDH(Reagent Kit	Swemed Diagnostics, India	LDH 25-125
Aspartate amino transferase (AST) Assay Kit	Agappe Diagnositics LTD, India	SGOT 11408005
Alanine Amino Transferase (ALT) Assay Kit	Agappe Diagnositics LTD, India	SGOT 11409005

Table S 2 : Antibodies

Antibody	Host	Clonality	Supplier	Catalog. No.
Beta Actin Monoclonal Antibody	Mouse	Monoclonal	Thermo Fisher Scientific, USA	MA1-140
Phospho-AMPK alpha-1 (Ser486) Polyclonal Antibody	Rabbit	Polyclonal	Thermo Fisher Scientific, USA	PA5-36615
AMPK alpha-1 Monoclonal Antibody	Mouse	Monoclonal	Thermo Fisher Scientific, USA	MA5-15815

Raw Western blot images

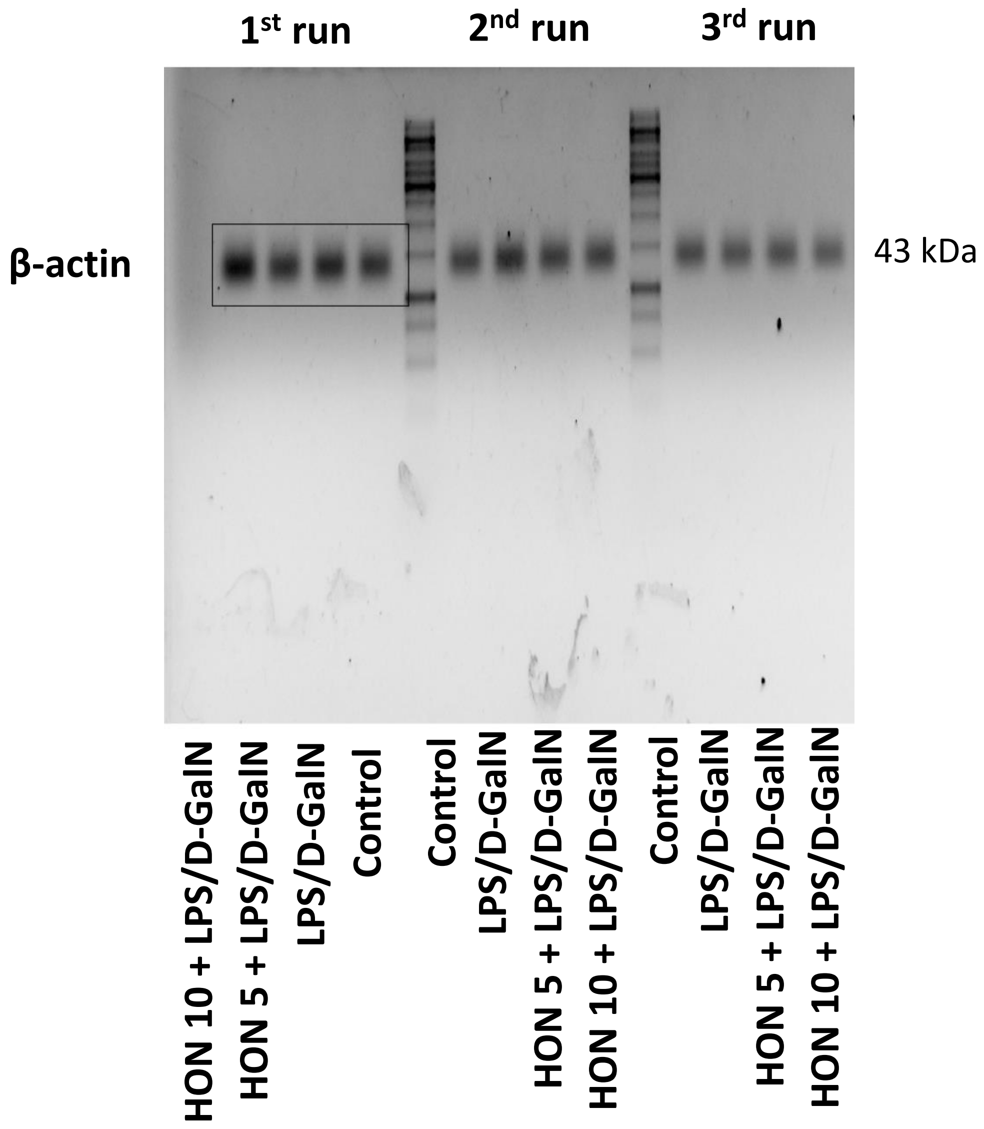

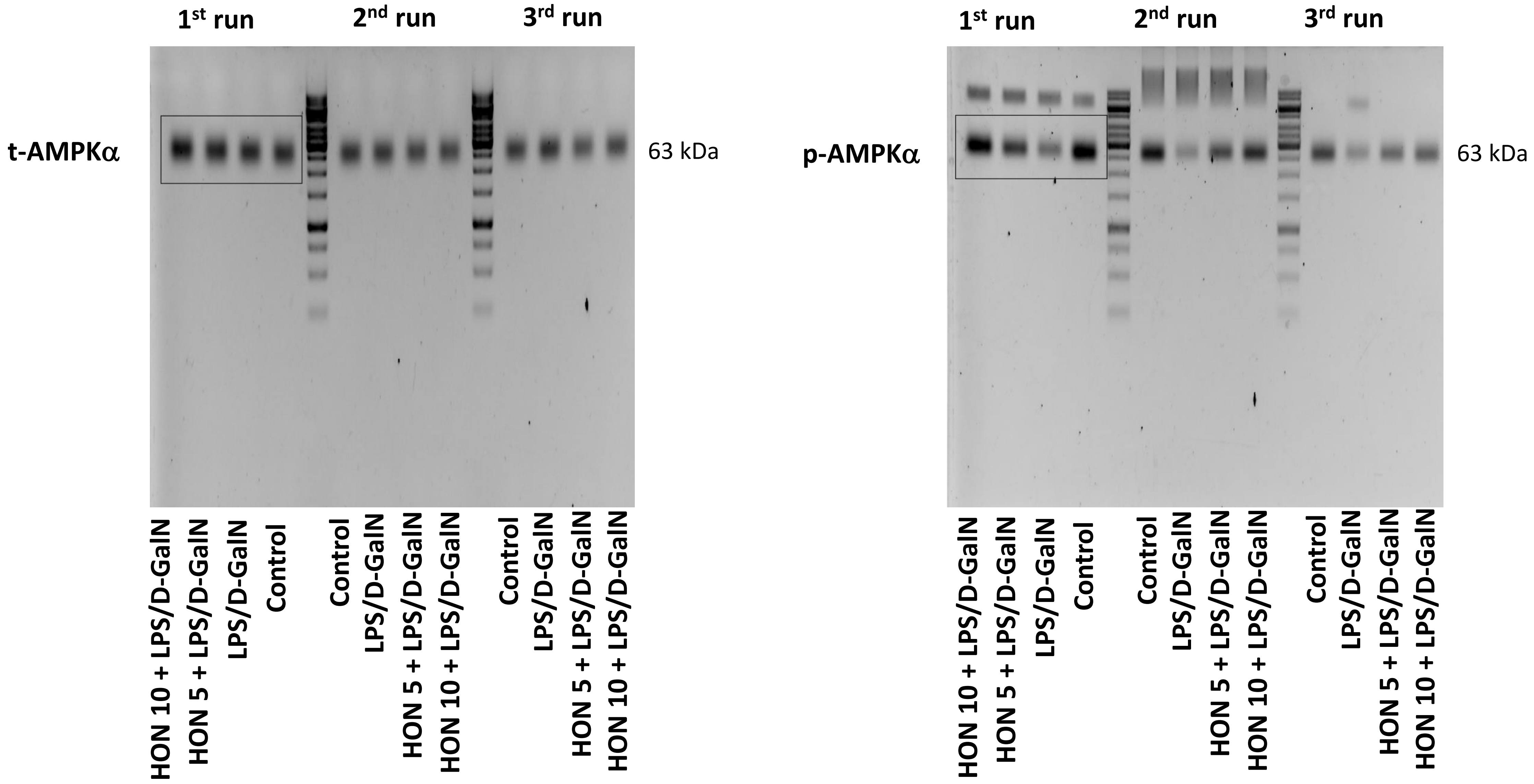
1st run
2nd run
3rd run
t-AMPKα
63 kDa
HON 10 + LPS/D-GalN
HON 5 + LPS/D-GalN
LPS/D-GalN
Control
Control
LPS/D-GalN
HON 5 + LPS/D-GalN
HON 10 + LPS/D-GalN
Control
LPS/D-GalN
HON 5 + LPS/D-GalN
HON 10 + LPS/D-GalN
1st run
2nd run
3rd run
p-AMPKα
63 kDa
HON 10 + LPS/D-GalN
HON 5 + LPS/D-GalN
LPS/D-GalN
Control
Control
LPS/D-GalN
HON 5 + LPS/D-GalN
HON 10 + LPS/D-GalN
Control
LPS/D-GalN
HON 5 + LPS/D-GalN
HON 10 + LPS/D-GalN
